# Shiga Toxin—A Model for Glycolipid-Dependent and Lectin-Driven Endocytosis

**DOI:** 10.3390/toxins9110340

**Published:** 2017-10-25

**Authors:** Ludger Johannes

**Affiliations:** Cellular and Chemical Biology Department, Institut Curie, PSL Research University, U1143 INSERM, UMR3666 CNRS, 26 rue d’Ulm, 75248 Paris CEDEX 05, France; ludger.johannes@curie.fr

**Keywords:** glycosphingolipid, globotriaosylceramide, Gb3, raft, galectin, integrin, CD44, cholera toxin, thermal Casimir-like force, spontaneous curvature

## Abstract

The cellular entry of the bacterial Shiga toxin and the related verotoxins has been scrutinized in quite some detail. This is due to their importance as a threat to human health. At the same time, the study of Shiga toxin has allowed the discovery of novel molecular mechanisms that also apply to the intracellular trafficking of endogenous proteins at the plasma membrane and in the endosomal system. In this review, the individual steps that lead to Shiga toxin uptake into cells will first be presented from a purely mechanistic perspective. Membrane-biological concepts will be highlighted that are often still poorly explored, such as fluctuation force-driven clustering, clathrin-independent membrane curvature generation, friction-driven scission, and retrograde sorting on early endosomes. It will then be explored whether and how these also apply to other pathogens, pathogenic factors, and cellular proteins. The molecular nature of Shiga toxin as a carbohydrate-binding protein and that of its cellular receptor as a glycosylated raft lipid will be an underlying theme in this discussion. It will thereby be illustrated how the study of Shiga toxin has led to the proposal of the GlycoLipid-Lectin (GL-Lect) hypothesis on the generation of endocytic pits in processes of clathrin-independent endocytosis.

## 1. Introduction

Shiga toxin is a pathogenic protein that is produced by *Shigella dysenteriae*, while enterohemorrhagic strains of *Escherichia coli* secrete Shiga-like toxins, which are also termed verotoxins [1]. Notably, the verotoxins of *E. coli* strain O157:H7 are responsible for pathological manifestations that can lead to hemolytic-uremic syndrome (HUS), the leading cause for pediatric renal failure in the world [2]. These toxins are also a threat to adults as it became apparent in 2011, when an outbreak with *E. coli* strain O104:H4 in Germany and neighboring countries claimed dozens of adult victims, and thousands of adult patients who were hospitalized with severe symptoms. The most life-threatening extra-intestinal disease manifestations are renal failure and central nervous system complications. To date, no specific treatment options exist, and clinical management of HUS remains purely supportive [3]. 

In the cytosol of target cells, the A-subunits of Shiga/verotoxins catalyze the deadenylation of position 4324 of 28S ribosomal RNA, leading to protein biosynthesis inhibition and subsequently to cell death. The A-subunits alone cannot enter cells, however. For this, they need the non-covalent interaction with receptor-binding homopentameric B-subunits. All of these toxins share a common cellular receptor: the neutral glycosphingolipid (GSL) globotriaosylceramide (Gb3 or CD77) [1].

**Figure 1:**
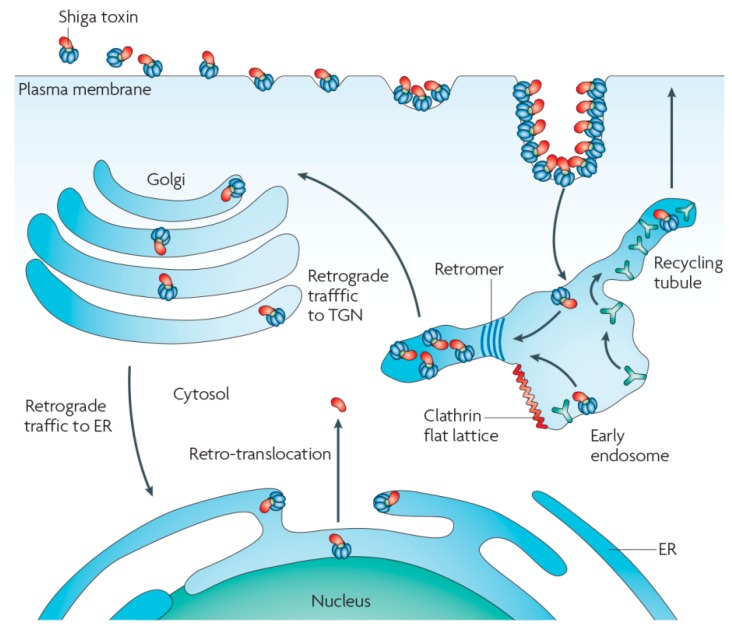
Intracellular trafficking of Shiga toxin. Reproduced from [1]. 2010, Nature Publishing Group.

Toxin trafficking from the plasma membrane of target cells to cytosolic ribosomes has been particularly well studied for Shiga toxin, on which this review will be focused with an emphasis on recent literature. The principal steps are receptor binding and toxin clustering at the plasma membrane of target cells, the formation of membrane invaginations, and tubular endocytic pits, the scission of these invaginations to form endocytic carriers, and their intracellular trafficking to endosomes and the retrograde route (Figure 1). These steps are discussed in the following sections of this review.

## 2. Receptor Binding and Toxin Clustering

The receptor-binding B-subunit of Shiga toxin (STxB) is a homopentameric protein of 35 kDa. Each STxB pentamer displays a total of 15 Gb3 binding sites [4], and each of these binding sites has only millimolar affinity for the globotriose sugar moiety of Gb3 (reviewed in Reference [5]). Yet, Shiga toxin binds avidly and strongly to cells. This apparent high affinity is the result of multiple bond interactions, one STxB pentamer being in contact with several Gb3 molecules at a time. To release STxB from the membrane, all of these interactions have to be dissociated at the same time, which is statistically unlikely. Such avidity effect is a general hallmark of lectins. For example, the homopentameric receptor-binding B-subunit of cholera toxin from *Vibrio cholerae* has a relatively low binding pocket affinity in the micromolar range for its cellular receptor, the GSL GM1 (reviewed in References [5,6]). Yet, much like Shiga toxin, cholera toxin also binds to cells with apparent nanomolar affinity, due to avidity interactions.

The avidity binding effect would be favored if the GSLs that function as toxin receptors were themselves clustered, such that several receptor binding sites on toxin pentamers would be occupied straight away upon the initial contact with the plasma membrane of target cells. Using an advanced electron microscopy technique, nanoclustering has indeed been documented for the gangliosides GM1 and GM3 [7]. This resembles the situation described for another class of glycosylated lipids, the glycosylphosphatidylinositol (GPI)-anchored proteins, which were equally described to exist in membranes as nanoclusters of a few molecules [8].

**Figure 2:**
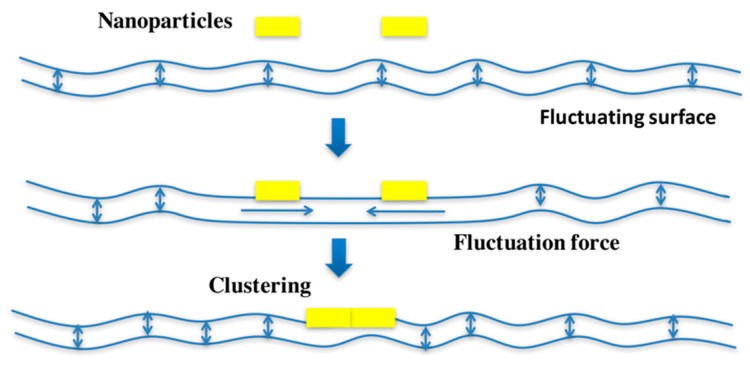
Hypothesis on fluctuation force-driven clustering. The represented nanoparticles could be Shiga toxin pentamers.

For Shiga toxin, no direct interaction between toxins has been detected. Yet, on model membranes [9] and on cells [10], Shiga toxin clusters readily, suggesting that a membrane-mediated mechanism drives toxin molecules together. A recent study has investigated this aspect in further detail. Based on theoretical modeling, computer simulations, and experiments in model membrane systems and on cells, an original hypothesis has been proposed [10] according to which Shiga toxin molecules would suppress thermally excited membrane fluctuations not only at sites at which they bind, but also on the membrane patch between 2 adjacent toxin molecules, as long as these are not further apart than roughly the size of the toxin itself (Figure 2). 

Unperturbed fluctuation of the membrane outside this toxin-delineated patch would push the toxin molecules together, even if these were not experiencing a direct attractive force. On theoretical grounds, such thermally induced membrane fluctuation forces (also termed thermal Casimir-like forces; References [11,12]; for a review, see Reference [13]) are expected to be as strong as electrostatic or van der Waals interactions, as long as the membrane inclusions that generate them are several nanometers in size [10]. However, as opposed to electrostatic or van der Waals interactions that operate at subnanometric distances, membrane fluctuation forces would have an effective radius of several nanometers, which corresponds to a gap in the interaction landscape of biological membranes between cytoskeleton-driven clustering that operates at tens to hundreds of nanometers [14], and the conventional subnanometric interaction forces. Of note, while membrane fluctuation forces are expected to apply to any tightly membrane-associated protein (or nanoparticle), other interaction forces would of course continue to be present in biological systems. Thereby, initial contacts that could be favored by the fluctuation forces would need to be further stabilized by other interactions for the generation of biologically meaningful outputs, and to avoid that all proteins (or nanoparticles) that are submitted to fluctuation forces coalesce into one big aggregate.

## 3. Formation of Membrane Invaginations and Tubular Endocytic Pits

Upon binding to Gb3, Shiga toxin does not directly reach through the plasma membrane to interact with the conventional cytosolic clathrin machinery. Yet, Shiga toxin has been seen in clathrin-coated pits [15], even if efficient inhibition of clathrin function causes at most a 35% reduction of Shiga toxin uptake [16,17]. How this localization into the clathrin pathway is operated remains to be further investigated. A key question here is whether toxin molecules “fall” into preexisting pits, or whether pit biogenesis is actually triggered by the toxin.

**Figure 3:**
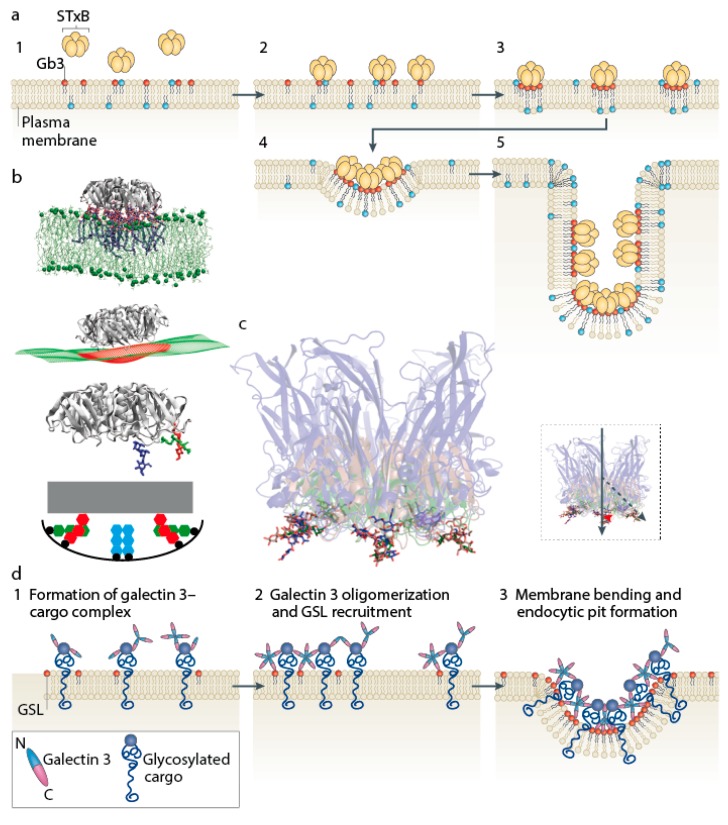
Lectin-driven construction of tubular endocytic pits. (**a**) Model for Shiga toxin B-subunit (STxB)-driven formation of membrane invaginations. (**b**) Molecular dynamics data on spontaneous curvature induced by STxB. The red and green binding sites represented in the lower part of the panel force the membrane to bend up at the edges of STxB pentamers. (**c**) Overlays of crystal structures of STxB (green, Reference [4]), cholera toxin B-subunit (red, Reference [20]), and VP1 capsid protein from SV40 (blue, Reference [21]). (**d**) GL-Lect hypothesis on the Gal3-driven, glycolipid-dependent formation of endocytic pits. Glycolipids are represented as red dots. Reproduced from [22]. 2015, Nature Publishing Group.

A sizable fraction of Shiga toxin still enters cells under conditions of clathrin pathway inhibition. In fact, protein toxins from plants and bacteria were the first cargo molecules for which clathrin-independent uptake into cells was suggested more than 35 years ago [18,19]. How membrane bending and endocytic pit formation are operated in these cases has remained a conundrum. Based on the observation that the formation of a Shiga toxin-Gb3 complex is necessary and sufficient to drive the formation of narrow tubular membrane invaginations [9], a molecular hypothesis has recently been suggested for how this might be achieved (Figure 3a). According to this model, the Shiga toxin-Gb3 complex would be endowed with curvature-active properties, i.e., the capacity to deform the membrane without the need of the cytosolic clathrin machinery. The clustering of several toxin molecules, likely favored by the fluctuation forces that were mentioned above, would then lead to the formation of deep and narrow invaginations as a first step towards the formation of tubular endocytic pits.

At first sight, this proposal appears counterintuitive. The asymmetric deposition of toxin molecules on the exoplasmic leaflet should lead to steric stress that would be expected to favor the buckling of the membrane to the outside of the cell or model membrane to which it binds, thereby generating positive curvature [23]. Obviously, the curvature-active properties of the Shiga toxin-Gb3 complex overcome this steric stress-induced buckling. How is this achieved? Results from molecular dynamics simulations have recently allowed to propose an explanation. It was shown that also in silico, the receptor-binding B-subunit of Shiga toxin (STxB) induces an increment of negative inward-oriented curvature when interacting with a patch of membrane that contains Gb3 receptor molecules [24] (Figure 3b). On average, 13 out of 15 Gb3 binding sites per STxB molecule were occupied. Because of the positioning of 10 of the Gb3 binding pockets at the rim of STxB molecules in a location slightly above the normal plane of the membrane, the latter must bend up to reach these sites, thereby generating an increment of negative, inward-oriented curvature.

This binding site geometry is preserved for the receptor-binding parts of cholera toxin and simian virus 40 (SV40) (Figure 3c), for which it was shown previously that they also have curvature-active properties, endowing them with the capacity to drive tubular membrane invaginations through interaction with their GSL receptor molecules [25], as observed for Shiga toxin [9]. Strikingly, these GSL-binding pathogenic lectins do not have any sequence similarity, which suggests that this binding site geometry might be the result of convergent evolution towards a common function: membrane mechanical work in relation to inward-oriented curvature generation for the construction of endocytic pits. Of note, cholera toxin and SV40 have indeed both been described to be efficiently internalized into cells in which the clathrin pathway is inhibited [18,26]. Further pathogens and pathogenic factors exist that also interact with GSLs in one way or another to get into cells (reviewed for gangliosides in Reference [27]), suggesting that this mechanism is used more widely. 

An example of a cellular lectin that drives the GSL-dependent biogenesis of tubular endocytic pits for the uptake of endogenous cargo proteins via clathrin-independent carriers has indeed been described: galectin-3 (Gal3). Gal3 is a member of a family of 15 galectins, 12 of which are expressed in human [28]. This galactose-binding lectin has many physiological (cell migration, immune modulation, inflammation, signaling, etc.) and pathological (cancer, fibrosis, diabetes, etc.) functions, raising the question of how a single protein can manage all of this. The cell biology of Gal3 still remains very little explored. Interestingly, it has recently been shown that membrane-associated Gal3 drives the GSL-dependent formation of narrow tubular invaginations on model membranes [29], similar to Shiga toxin [9], cholera toxin, and SV40 [25]. On cells, Gal3 drives the GSL-dependent biogenesis of clathrin-independent carriers (CLICs), via which Gal3-interacting proteins such as the cell adhesion and migration factors CD44 and β1 integrin are internalized [29].

Based on the results published in Reference [29], the GlycoLipid-Lectin (GL-Lect) hypothesis was put forward [22]. According to this hypothesis, monomeric Gal3 in solution binds to glycosylated cargo proteins such as CD44 and β1 integrin on target cells (Figure 3d). This leads to the oligomerization of Gal3 and its capacity to interact with GSLs in a way such as to induce membrane bending and thereby, the biogenesis of endocytic pits, from which CLICs are then generated. According to this model, Gal3 functions like an endocytic adaptor that links glycosylated cargo proteins and membrane curvature-generating GSLs into the same compositional nanoenvironments from which tubular endocytic pits emerge. In contrast, the pathogenic lectins from Shiga toxin, cholera toxin, and SV40 are themselves cargoes, which are produced as stable pentamers (Figure 3c) such that they can immediately aim for the membrane mechanical part of the program, bypassing the cargo recognition step.

Several aspects of the GL-Lect model are worth further discussion. One concerns the question of why monomeric Gal3 fails to bind GSLs directly. Most likely, the affinity of the sugar binding pocket of Gal3 for carbohydrates on GSLs is weak, such that efficient binding becomes possible only upon the formation of multiple-bond interactions (avidity) of each Gal3 oligomer with several GSLs. Another key question concerns the membrane-bound structure of Gal3. The C-terminal carbohydrate recognition domain of the protein has been crystallized in solution, while the N-terminal domain turned out to be unstructured [30]. It is still unknown when and how the N-terminal domain folds, and what type of oligomeric configuration Gal3 adopts on membranes. It has been proposed that the protein may form pentamers [31], but also, that even larger oligomers may arise [32]. One may finally point out that GSLs are basic fabric of raft-type membrane domains [33]. The lectin-induced clusters of GSLs (or of other glycolipids such as GPI-anchored proteins) that are likely formed at sites of CLIC formation might therefore be viewed as stabilized raft-type membrane domains [34], whose intrinsic connectivity may be critical for tubular endocytic pit construction, CLIC formation, and subsequent intracellular sorting.

## 4. Membrane Scission, Targeting to Endosomes, and Retrograde Transport

Shiga toxin-induced membrane invaginations need to pinch off from the plasma membrane by fusion of the opposing walls of invaginated tubular endocytic pits. The conventional pinchase dynamin affects Shiga toxin uptake, but is not absolutely required [9,16]. The difficulty with coming to a conclusion for the involvement of dynamin is that feedback loops exist to actin [35], which also contributes to the scission of Shiga toxin-induced membrane invaginations [36]. It has indeed been demonstrated that these invaginations are prone to undergo domain formation, thereby generating domain boundary forces that drive the spontaneous line tension-driven squeezing of the tubule membrane leading to scission [37]. Actin was identified as one of the domain formation triggers [36], likely by directly or indirectly binding to lipids, thereby changing the entropy of the system [38].

**Figure 4:**
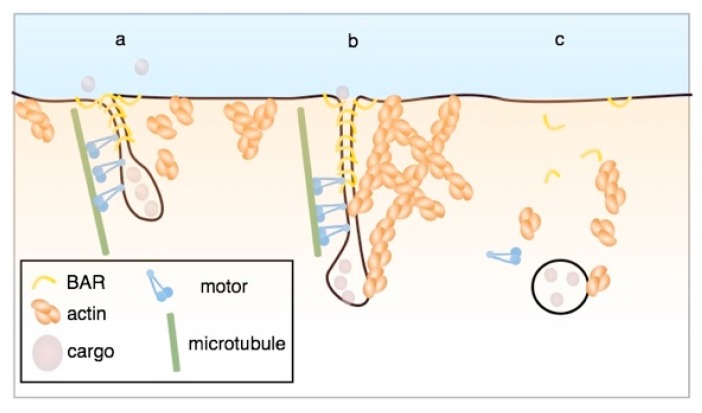
Friction-driven scission. See text for details. Reproduced from [39]. 2017, Cell Press.

Recent findings indicate that yet another scission modality might be operating on Shiga toxin-induced membrane invaginations. The model that is presented in Figure 3a predicts that the signal that is sent by the toxin to the cytosol might be mechanical: the formation of a highly curved membrane domain, which would then be recognized by cellular machinery for further processing (Figure 4).

Proteins of the Bin, amphiphysin, and Rvs (BAR) domain family are specialized in such curvature recognition [40], and by screening through a BAR domain protein library, endophilin-A2 (EndoA2) was identified as being functionally localized onto Shiga toxin-induced membrane invaginations in relation to the scission reaction [41]. Furthermore, it was demonstrated for Shiga [41] and cholera toxin [42] that these invaginations are pulled upon by microtubule-based dynein motor activity. Strikingly, if this dynein-mediated pulling force is exerted on tubular invaginations that are scaffolded by EndoA2, membrane scission ensues [41]. This is due to the fact that the EndoA2 scaffold exerts a friction force onto the membrane that limits lipid diffusion [39]. Upon pulling with speeds superior to 50 nm/s (such as that exerted by the dynein motor), the lack in lipid supply leads to tube thinning, and then to scission. Such pulling force or friction-driven scission may operate more generally in processes of clathrin and caveolin-independent endocytosis that are often little reliant on dynamin [43].

It might appear surprising that several scission modalities operate on Shiga toxin-induced membrane invaginations: dynamin [9], actin [36], and EndoA2/dynein [41]. It could be demonstrated that when interfering with each of these individually or in combination, the effects on scission appear to be roughly additive [41], suggesting that the overall probability of scission is the result of individual contributions.

Once formed, Shiga toxin-containing carriers are targeted to the endosomal system. This is favored by association with microtubules [44], which might be initiated right at the plasma membrane, as discussed above. Furthermore, the vSNARE proteins VAMP2, VAMP3, and VAMP8 are recruited to Shiga toxin-containing endocytic carriers for their targeting to the endosomal membrane system [45].

From endosomes, Shiga toxin is transported via the retrograde route to the TGN, the Golgi apparatus, and then on to the endoplasmic reticulum, from where the catalytic A-subunit is translocated to the cytosol [46]. The study of Shiga toxin has allowed to propose the existence of a trafficking interface between early endosomes and the TGN [47], which has since become a hotspot of membrane biology research. Shiga toxin trafficking at this interface has been described to depend on Rab6 [48], SNARE complexes involving syntaxin-16 [48,49] and syntaxin-5 [50], epsinR [17], Arl1 [51], OCRL [52], retromer [53,54,55], the tethering complex GARP [56] and the GARP interactor TSSC1 [57], the ARF1 GAP protein AGAP2 [58], GPP130 [59], the ERM proteins ezrin and moesin [60], annexins A1 and A2 [61], and UNC50 [62].

The early endosome-TGN trafficking interface has also become the target of small molecule inhibitors to protect against the Shiga toxin [63,64]. The Retro series compounds have been shown to protect cells and mice against Shiga toxin [65] and the plant toxin ricin that also uses the retrograde transport route to intoxicate cells [66]. In Retro compound-treated cells, Shiga toxin is retained in early endosomes, therefore reaching the endoplasmic reticulum and the cytosol less efficiently, leading to a reduced toxic effect. Efforts to identify the cellular target(s) of these compounds are still ongoing.

## 5. Conclusions

The study of Shiga toxin endocytosis has allowed the discovery of a wide range of novel cellular mechanisms that had previously gone undetected. Yet, much remains to be done. Notably the functional dissection of the Shiga toxin receptor, the GSL Gb3, is still in its infancy, due to the difficulty to manipulate the structure of this glycosylated lipid in a controlled manner within cells. This is true for GSLs in general, notably for the ones with long acyl chains that are found in naturally occurring species and that are very difficult to reconstitute into cells. Several of the mechanisms that were discovered with the help of Shiga toxin are themselves still poorly explored. To name two examples: Fluctuation forces for the clustering of tightly membrane-associated proteins (or nanoparticles) will need to be measured directly using appropriate force sensors, and the structural intricacies underlying the capacity of GL-Lect drivers such as galectin-3 (Gal3) to switch from glycoprotein to glycolipid recognition remain to be elucidated. Membrane biology research on or around Shiga toxin is thereby expected to remain a fruitful ground for discovery.
